# Further delineation of Loeys-Dietz syndrome type 4 in a family with mild vascular involvement and a *TGFB2* splicing mutation

**DOI:** 10.1186/s12881-014-0091-8

**Published:** 2014-08-28

**Authors:** Marco Ritelli, Nicola Chiarelli, Chiara Dordoni, Stefano Quinzani, Marina Venturini, Roberto Maroldi, Piergiacomo Calzavara-Pinton, Marina Colombi

**Affiliations:** 1 Division of Biology and Genetics, Department of Molecular and Translational Medicine, Medical Faculty, University of Brescia, Viale Europa 11, Brescia 25123, Italy; 2 Department of Dermatology, University Hospital Spedali Civili, Brescia, Italy; 3 Department of Radiology, University of Brescia, Brescia, Italy

**Keywords:** Loeys-Dietz syndrome type 4, TGFB2, TGF-ß2, Splicing mutation

## Abstract

**Background:**

The Loeys-Dietz syndrome (LDS) is a rare autosomal dominant disorder characterized by thoracic aortic aneurysm and dissection and widespread systemic connective tissue involvement. LDS type 1 to 4 are caused by mutations in genes of the TGF-β signaling pathway: *TGFBR1* and *TGFBR2* encoding the TGF-β receptor (LDS1 and LDS2), *SMAD3* encoding the TGF-β receptor cytoplasmic effector (LDS3), and *TGFB2* encoding the TGF-β2 ligand (LDS4). LDS4 represents the mildest end of the LDS spectrum, since aneurysms are usually observed in fourth decade and the progression of the disease is slower than in the other forms.

**Case presentation:**

We report the clinical and molecular findings of an LDS4 Italian family. Genetic testing included *TGFBR1*, *TGFBR2*, *SMAD3*, and *TGFB2* analysis by Sanger sequencing. In order to verify the effect of the identified splice mutation, RT-PCR analysis was performed.

The proband, a 57-year-old woman, showed high palate, hypoplasic uvula, easy bruising, joint hypermobility, chronic pain, scoliosis, multiple relapsing hernias, dural ectasia, and mitral valve prolapse. Magnetic resonance angiography revealed tortuosity and ectasia of carotid, vertebral, cerebral, and segmental pulmonary arteries. Arterial aneurysm and dissection never occurred. Her 39- and 34-year-old daughters presented with a variable degree of musculoskeletal involvement. Molecular analysis disclosed the novel c.839-1G>A splice site mutation in the *TGFB2* gene. This mutation activates a cryptic splice acceptor site in exon 6 leading to frameshift, premature termination codon and haploinsufficiency (p.Gly280Aspfs*41).

**Conclusions:**

Our data confirm that loss-of-function mutations in *TGFB2* gene do not always lead to aggressive vascular phenotypes and that articular and skeletal signs are prevalent, therefore suggesting that LDS4 must be considered in patients with sparse signs of LDS and related disorders also in the absence of vascular events.

## Background

Thoracic Aortic Aneurysms and Dissections (TAADs) are a common cause of sudden death of young adults. The pathophysiology of TAAD is complex and multi-factorial. To date, though classic cardiovascular risk factors play a pivotal role in a majority of patients, several genes have been identified in both syndromic and nonsyndromic forms of TAAD, including *FBN1* (Marfan syndrome, MFS) [1], *TGFBR1*, *TGFBR2*[2],[3] and *SMAD3* (Loeys-Dietz syndrome type 1 to 3, LDS) [4], *SLC2A10* (Arterial tortuosity syndrome, ATS) [5], *ACTA2* (TAAD with *livedo reticularis* and *iris flocculi*) [6], *MYH11* (TAAD with *patent ductus arteriosus*) [7], and *MYLK* (TAAD7) [8]. Although clinical features show significant overlap, these entities differ in the extent of vascular involvement and clinical course and, as a consequence, molecular characterization has become crucial in the evaluation, counseling and management of these patients. Among the syndromic forms of TAAD, LDS is a rare autosomal dominant disorder with widespread systemic involvement and connective tissue disorders (CTD) signs. LDS type 1 (LDS1, MIM**#**609192) and type 2 (LDS2, MIM**#**610168) are caused by heterozygous mutations in the genes encoding the TGF-ß receptors type 1 and 2, which bind TGF-β and initiate cellular signaling [2],[3]. The majority of individuals with LDS1 and 2 shows vascular findings with life-threatening complications, such as aortic dissection and widespread aortic aneurysms, and life expectancy is reported to be 37 years [9],[10]. Craniofacial signs as ocular hypertelorism, bifid uvula/cleft palate or craniosynostosis and cutaneous manifestations, as velvety and translucent skin, easy bruising, atrophic scars, are also present at variable degree [2],[3],[9],[11]. In the first reports, LDS patients were stratified into 2 types, depending on severity of craniofacial features (type 1) or cutaneous features (type 2). However, these findings are now believed to be part of a continuum within the LDS spectrum of disease [12]. Mutations in the *SMAD3* gene, which encodes a protein critical for cellular signaling downstream of the TGF-β receptors after ligand binding, are responsible for LDS type 3 (LDS3, MIM#613795), also known as Aneurysms–Osteoarthritis Syndrome. LDS3 patients share phenotypic manifestations with LDS1 and 2, i.e., aortic root aneurysm, vascular dissection, skeletal deformities, and typically show osteoarthritis at young age [13],[14], although families without osteoarthritis are also reported [15].

Lindsay et al. and Boileau et al. identified an additional gene responsible for familial TAAD, the *TGFB2* gene, which encodes the ligand TGF-β2 [16],[17]. In particular, Boileau et al. discovered heterozygous mutations in 2 families and 2 sporadic cases with TAA [17], and Lindsay et al. identified *TGFB2* mutations in 8 families [16]. Both groups examined aortic tissue from probands and found fragmentation of elastin fibers and deposition of collagen and proteoglycans, similar to what is observed in tissues from MFS and LDS patients. Furthermore, altered TGF-β signaling, as judged by an increase in TGF-β1 or TGF-β2 protein expression and greater nuclear accumulation of phosphorylated SMAD2, was demonstrated. In *TGFB2* heterozygous knockout (*Tgfb2*^+/−^) mice Lindsay et al. found aortic root dilatation and increased TGF-β signaling, consistent with the human disease presentation [16]. Careful clinical phenotyping of *TGFB2* mutation-bearing individuals identified a variety of vascular, musculoskeletal, and other features shared by patients with either MSF or LDS, therefore, the disorder was classified as LDS4 (MIM**#**614816). Features shared with MFS and LDS include aortic aneurysm, mainly in thoracic tract, *pectus* deformity, arachnodactyly, scoliosis, joint hypermobility, and *striae distensae* in young age. Features shared with LDS, but not MFS, include hypertelorism, bifid uvula, bicuspid aortic valve, mitral valve prolapse (MVP), arterial tortuosity, club feet, and thin skin with easy bruising [16]. In LDS4 aortic aneurysm presents at later onset; relatively mild aortic dilatation, mainly at the level of the sinus of Valsalva, and a lower incidence of dissection compared to the other LDS types was observed, and the reported mean age of aortic disease was 35 years [17]. Renard et al. described other 6 sporadic LDS4 patients with *TGFB2* mutations, pointing out a high rate of mitral valve disease, suggesting that this might be a signature feature of the disorder that may direct molecular analysis [18]. Leutermann et al., very recently described another LDS4 family with three affected members [19]. Recently a revised LDS nosology was proposed, in particular the authors suggested that a mutation in any of these 4 genes in combination with documented aneurysms or dissection should be sufficient for the diagnosis of LDS [20].

Here, we report on a new LDS4 family with a 57-year-old proband without thoracic aortic aneurysms and other major vascular complications, caused by a novel mutation in the *TGFB2* gene and compare the clinical features of the affected members with those reported in the literature. Our data suggest that LDS4 must be considered in patients with sparse signs of LDS and related disorders, also in the absence of vascular events.

## Case presentation

### Patients, materials and methods

We describe an Italian LDS4 family with three affected members: the proposita and her 34-year-old daughter, underwent careful clinical evaluation and provided written informed consent for genetic testing. The clinical features of the 39-year-old daughter were reported by the proposita, since this daughter did not consent clinical evaluation and genetic testing, likewise both proband’s parents, in the octave decade and with a referred unremarkable clinical history, could not be evaluated.

The proposita underwent extensive cardiovascular studies, including electrocardiogram (ECG), echocardiography, and brain, thoracic and abdominal magnetic resonance angiography (MRA). The 34-year-old daughter did not consent MRA study and only ECG and echocardiography with aortic root evaluation were performed. Genomic DNA was purified from blood samples using Wizard Genomic DNA purification Kit (Promega) by standard procedures. Total RNA was isolated from proband’s whole blood using NucleoSpin^®^ RNA Blood Midi Kit (Macherey-Nagel) and retrotranscribed in the presence of random hexanucleotide primers and MMLV-RTase (Life Technologies) by standard procedures. Primers were designed for all coding exons, including their intron–exon boundaries, and for the 5′ and 3′ UTRs near the coding sequence (a minimum of 200 bp), using the Primer3 tool (http://frodo.wi.mit.edu/). Primer sequences were checked for the absence of known variants, such as single nucleotide polymorphisms, based on dbSNP version 139 (www.ncbi.nlm.nih.gov/projects/SNP/). In particular, all exons and intron flanking regions of *TGFBR1*, *TGFBR2, SMAD3* and *TGFB2* genes were amplified by standardized PCR using optimized genomic primer-sets and the GoTaq Ready Mix 2X (Promega) (primer sequences and amplification profiles are available upon request). After enzymatic cleanup of the PCR products by ExoSap-IT^®^ (Affymetrix), all fragments were sequenced in both orientations using the BigDye^*®*^ Terminator Cycle Sequencing kit protocol (Life Technologies) and the Performa^®^ DTR Ultra 96-Well Plates (EdgeBio) for PCR cleanup followed by capillary electrophoresis on the ABI3130XL Genetic analyzer (Life Technologies). The identified *TGFB2* mutation was not found in 100 control individuals or in the 1000 Genomes Project or NHLBI Exome Variant Server (ESP6500) databases. Nucleotide and protein accession numbers correspond to the *TGFB2* (NM_000093.3, NP_000084.3) reference sequences. The mutation was annotated according to HGVS nomenclature (www.hgvs.org/mutnomen), and the sequence description verified using Mutalyzer (www.LOVD.nl/mutalyzer). Nucleotide numbering was based on cDNA sequence numbering with +1 corresponding to the A of the ATG translation initiation codon 1 in the reference sequence. For protein numbering, +1 corresponds to the first translated amino acid. The sequences were analyzed using the Sequencher 4.9 software (www.genecodes.com). To evaluate the identified splice site mutation, we used four prediction programs (SpliceSite-Finder-like, MaxEntScan, NNSPLICE and Human Splicing Finder) in the Alamut Software version 2.4 (www.interactive-biosoftware.com). Further, to verify the effect of the splice mutation, RT-PCR was carried out by standard procedures; in particular, amplification of cDNA covering exons 5–7 of the *TGFB2* gene was performed.

### Clinical findings

**Figure 1:**
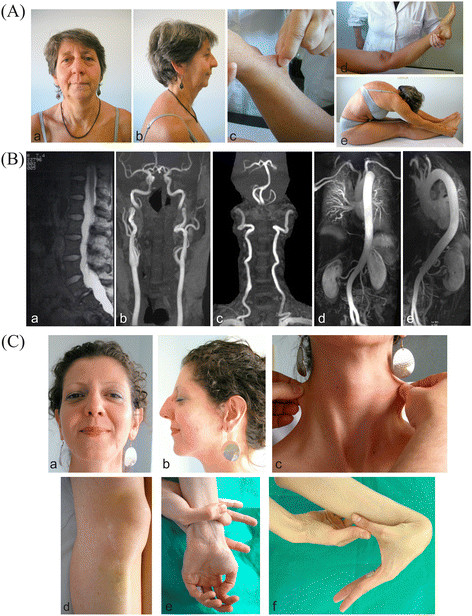
**Clinical and radiological features of the proband and her daughter. (A)** Clinical phenotype of the proband including microretrognathia, and elongated philtrum (a, b), mild generalized skin hyperextensibility (c), joint hypermobility (d, e). **(B)** MRI in the proposita showed dural ectasia of the lumbar tract (a), MRA disclosed tortuosity and ectasia of carotid, vertebral, and cerebral arteries (b, c) and marked tortuosity of both segmental pulmonary arteries (d). **(C)** Clinical presentation of the 34 years-old proband’s daughter: hypotelorism, elongated philtrum and malar hypoplasia (a, b), mild skin hyperextensibility over the neck (c), atrophic post-surgical scar and ecchymosis (d), arachnodactyly with positive wrist and thumb signs (e, f).

The proposita (Figure 1A) was a 57-year-old woman with family history remarkable for CTD in her two 39- and 34-year-old daughters. She was born from unrelated Italian parents after an uneventful pregnancy and delivery. Her perinatal and psychomotor development was normal. Two pregnancies resulted in the birth of two daughters. In the first pregnancy she reported premature membrane rupture at 34 weeks, and in the second a threatened abortion. The spontaneous menopause was at 50 years. Clinical history presented widespread signs of CTD. Since the childhood sub/luxations of the shoulders, wrists, knees and ankles were occurring. She suffered from acute articular rheumatism, diagnosed at 9 years, and right relapsing inguinal hernia, surgically treated at 9, 25, and 40 years. Since her twenties, she referred chronic generalized articular pain, mainly affecting her back, treated with NSAIDs. Magnetic resonance imaging (MRI), performed at 42 years, revealed dural ectasia, lumbar discal hernias (L5-S1) and hypoplasia of the twelfth ribs (Figure 1B). Clinical history also included crural hernia, surgically treated at 25 and 40 years, hiatal hernia with gastroesophageal reflux, chronic headache, gingival fragility, and easy bruising. Ectopia lentis was excluded by ophthalmologic evaluation. ECG and echocardiography, performed at 49 years for tachycardia, discovered paroxysmal supraventricular tachycardia and MVP with minimal regurgitation and normal systolic function (EF 65%). Following this analysis she underwent cryoablation therapy. At this age the aortic root diameter was normal. On examination at 56 years, she presented with normal stature (1,63 m), light blue sclerae, high arched palate, micrognathia, elongated *philtrum*, hypoplasic uvula, doughy and hyperextensible skin over the neck, the forearm, and the elbows, old aging aspect, *striae distensae* over the hips, joint hypermobility according to Beighton score (9/9), and scoliosis (Figure 1A). In a clinical suspicion of LDS, a brain, thoracic and abdominal MRA was scheduled, revealing tortuosity and ectasia of carotid, vertebral, and cerebral arteries, and marked tortuosity of two segmental pulmonary arteries (Figure 1B). No other vascular abnormalities were detected.

The proband reported that her first daughter presented tall stature (1,76 m) for the familial target of 1,58 m, hypotonia at birth, congenital *talipes*, umbilical hernia, conservative treatment for scoliosis with orthopedic corset at 13 years, chronic articular pain, recurrent dislocations, flat feet, hallux valgus needing surgery, easy bruising, and varicose veins. On evaluation at 34 years, the second daughter presented tall stature for the familial target (1,78 m), high arched palate, myopia, astigmatism, mild hyperextensible skin over the neck and the elbows, atrophic post-surgical scar over the knee, arachnodactyly, mild *pectus excavatum*, scoliosis, joint hypermobility according to the Beighton score (6/9) (Figure 1C). She referred recurrent right patella dislocations, meniscus and lateral ligaments tear requiring arthroscopic surgery in her youth, myopia, astigmatism, easy bruising and Hashimoto thyroiditis. An ECG and echocardiography revealed normal aortic root diameter, therefore excluding ectasia or aneurysm at aortic root level. Valvular system and systolic function (EF 75%) were normal, except for MVP with minimal regurgitation.

### Molecular characterization

**Figure 2:**
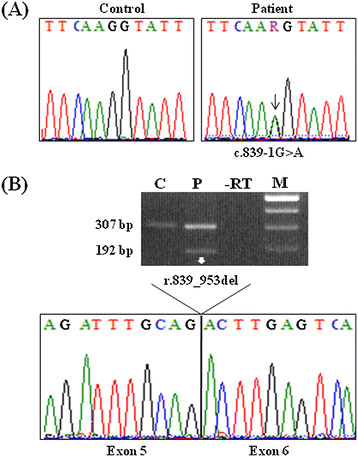
**Molecular characterization of the proband. (A)** The sequence analysis of the *TGFB2* gene disclosed the novel c.839-1G>A transition (arrow), affecting the splice acceptor site of exon 6. **(B)** Characterization of the splicing effect of the c.839-1G>A mutation: agarose gel electrophoresis of the cDNA, amplified with primers encompassing exons 5–7, showed in the patient (P) the presence of an aberrant band (192 bp), in addition to the wild type fragment (307 bp), which was also detected in the control (C). The sequencing of the aberrant PCR product showed the deletion of the beginning 115 bp of exon 6 (r.839_953del), demonstrating that c.839-1G>A mutation leads to the activation of a new cryptic splice acceptor site between cDNA nucleotides 953 and 954.

After written informed consent of the proband, mutational screening of the four LDS causative genes, i.e., *TGFBR1*, *TGBFR2*, *SMAD3* and *TGFB2,* was performed. This analysis revealed the presence of the novel c.839-1G>A splice mutation in intron 5 of the *TGFB2* gene, thus confirming the diagnosis of LDS4 (Figure 2A). The causal mutation was also found in the 34-year-proband’s daughter. All of the splicing evaluation programs of the Alamut software predicted that the c.839-1G>A mutation abolishes the canonical splice acceptor site. To verify the effect of the splicing mutation, RT-PCR analysis was performed on RNA purified from proband’s blood. Amplification of the cDNA region covering exons 5–7 demonstrated that the c.839-1G>A mutation leads to the activation of a cryptic splice acceptor site 115 bp downstream of the consensus site, generating a frameshift and a premature termination codon formation (PTC) in exon 7 (p.Gly280Aspfs*41) (Figure 2B). Furthermore, because of the lower amount of the aberrant band, compared to the wild type transcript, it is likely that the c.839-1G>A mutation activates to a certain degree the nonsense mediated RNA decay (NMD) pathway, finally resulting in TGF-β2 haploinsufficiency (Figure 2B).

### Discussion

**Figure 3:**
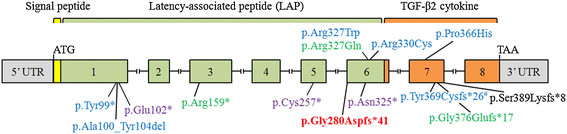
**Overview of currently known*TGFB2*mutations.** Exons and untranslated regions (UTR) are represented by numbered boxes. The signal peptide is represented by a yellow, the Latency-Associated Peptide (LAP) domain by a green, and the TGF-β2 cytokine domain by an orange box. Frameshift/truncating mutations are listed at the bottom, while missense mutations are listed on the top. The mutations reported by Boileau et al. [17] are in violet, those described by Lindsay et al. [16] in blue (^#^ also reported by Boileau et al. [17]), those in Renard et al. [18] in green, the mutation reported in Leutermann et al. [19] is in black, and the mutation identified in this study is in red. Numbering is based on transcript NM_001135599.2 and protein reference sequence NP_001129071.1.

LDS4 is a recently delineated autosomal dominant condition presenting with aneurysms, dissections, and tortuosity throughout the arterial tree in association with mitral valve disease, mild craniofacial features, and skeletal and cutaneous anomalies [16]–[19]. Mutations in the *TGFB2* gene accounted for 25% in Lindsay et al., 1.5% in Boileau et al., 4% in Renard et al., and 1.1% in Leutermann et al., of sampled familial cases of thoracic aortic disease that were not attributed to other known TAAD-causing genes [16]–[19]. So far, clinical data on LDS4 are available in a limited number of cases, i.e., 11 families and 8 sporadic patients all carrying *TGFB2* mutations (Table 1 and Figure 3). Altogether, 15 independent mutations were identified, 11 of which were whole-gene deletions, frameshifts or nonsense mutations that were predicted to cause to a certain degree the degradation of the cognate mRNA by NMD, and 4 were missense substitution of highly conserved amino acids with a predicted pathologic effect. In the family here described, a novel splicing mutation c.839-1G>A in intron 5 was identified in the proband and in her daughter. Amplification of the cDNA region covering exons 5–7 demonstrated that the mutation leads to the activation of a cryptic splice acceptor site within exon 6, that generates a PTC and activates to a certain level the NMD pathway. As such, the pathogenic mechanism underlying all the *TGFB2* mutations is most likely loss-of-function.

**Table 1:** **Overview of currently known** ***TGFB2*** **mutations**

**Exon**	^**a**^**c-notation**	^**b**^**p-notation**	**Domain**	**Reference**
1	c.294_308del	p.Ala100_Tyr104del	LAP	[16]
1	c.297C > A	p.Tyr99*	LAP	[16]
1	c.304G > T	p.Glu102*	LAP	[17]
3	c.475C > T	p.Arg159*	LAP	[18]
5	c.771C > A	p.Cys257*	LAP	[17]
**6**	**c.839-1G > A**	p.Gly280Aspfs*41	**LAP**	**Current study**
6	c.957_972dup	p.Asn325*	LAP	[17]
6	c.979C > T	p.Arg327Trp	LAP	[16],[18]
6	c.980G > A	p.Arg327Gln	LAP	[18]
6	c.988C > T	p.Arg330Cys	LAP	[16]
7	c.1097C > A	p.Pro366His	Cyt	[16]
7	c.1106_1110del	p.Tyr369Cysfs*26	Cyt	[16],[17]
7	c.1125del	p.Gly376Glufs*17	Cyt	[18]
7	c.1165dupA	p.Ser389Lysfs*8	Cyt	[19]
Entire gene Chr1.hg19: g.(215,588,712)_(222,145,072)del				[16]
Entire gene Chr1.hg19: g.(216,672,181)_(220,202,575)del				[16]

^**a**^DNA mutation numbering is based on the cDNA sequence. For cDNA numbering, +1 corresponds to the A of the ATG translation initiation codon in the reference sequence. The reference sequence is based on the GenBank Accession number: *TGFB2* NM_001135599.2. ^**b**^For protein numbering, +1 corresponds to the first translated amino acid. The reference sequence is based on the GenBank Accession number: *TGFB2* NP_001129071.1. *Abbreviations*: *LAP* Latency-associated peptide domain, *Cyt* TGF-β2 cytokine domain.

Comparable to several other conditions known as TGF-β vasculopathies and presenting with arterial aneurysms, such as MFS [21], the other LDS types [9] and ATS [5], *TGFB2* loss-of-function resulted in a activation in TGF-β signaling pathway [22],[23], as shown by the increase of phosphorylated SMAD2 and SMAD3 (SMAD2/3) levels in aortic lesions from *TGFB2*^+/−^ patients and *Tgfb2*^+/−^ mice, as well as by elevated ligand levels of either TGF-β1 [16] or TGF-β2 [17].

Up to date a high incidence of TAA or ectasia, mainly at the aortic root level, has been reported in LDS4 (Table 2). In particular, in Boileau et al. aortic root aneurysm was detected in 74% of the cases (14/19) [17], in Linsday et al. one main pulmonary artery aneurysm was reported; but a Z-score >2 was detected in 93% of the cases (14/15) [16]. In Renard et al. TAAs were present in 67% of the cases (4/6), and in Leutermann et al. aneurysms were present in two of the family members [18],[19]. Despite this high incidence of TAA or ectasia, a lower rate and later onset of aortic dissection is noted in LDS4 compared to other LDS types [9]. Considering the LDS4 patients reported so far, an overall dissection rate of 12% (5/43), at a median age of 46 years, emerged. Specifically, in Boileau et al. the aortic dissection rate was 11% (2/19 at 50 and 33 years), in Linsday et al. it was 7% (1/15 at 42 years), and in Renard et al. 33% (2/6, at 60 and about 46 years) [16]–[18]. None of the clinically evaluated members of the family described by Leutermann et al. had aortic dissections, but the proband (at 51 years) and his brother (at 47 years) underwent aortic aneurysm surgery and the index patient’s father died at 52 years after acute aortic dissection [19]. Valve sparing aortic root replacement (VSARR) for aortic root diameter between 45 and 56 mm was performed also in 20% of cases (3/15) in Linsday et al., at a median age of about 41 years [16]. Cerebral arteries involvement was delineated only in few cases; in particular, in Boileau et al. an anamnestic cerebrovascular disease was reported in 30% of the cases (3/10). Specifically, one episode of anterior communicating artery aneurysm rupture and two internal carotid aneurysm dissections were described; in the other 7 patients, investigated for neck and brain arteries, no aneurysm was detected [17]. In Renard et al. a severe cerebrovascular disease was documented in one patient, a 53-year-old woman who suffered from recurrent TIA, since she was 18-year-old, recurrent strokes and presence of tortuous vertebral arteries [18]. Lindsay et al. and Leutermann et al. did not report cerebrovascular events [16],[19].

**Table 2:** **Comparison of clinical findings between the current study and those of Boileau et al.**[17]**, Lindsay et al.**[16]**, Renard et al.**[18]**, and Leutermann et al.**[19]

			**Frequency**^**a**^				
**Clinical features**	**Proband**	**Daughter**	**Boileau et al.[**[17]**]**	**Lindsay et al.[**[16]**]**	**Renard et al.[**[18]**]**	**Leutermann et al.[**[19]**]**	**Total/percentage**
***Cardiovascular***							
TAA or MPA	-	-^**c**^	14/19	1/4 (11)	4/6	2/2 (1)	21/32 (13) 65%
Aortic dissection	-	-	2/19	1/15	2/6	0/3	5/45 11%
Aortic repair	-	-	4/19	3/15	0/6	2/3	9/45 20%
Arterial tortuosity	+	N.A.	3/5 (14)	1/4 (11)	1/1 (5^**d**^)	2/2 (1)	8/13 (32) 61.5%
MV impaired	+	+	3/19	7/10 (5)	4/6	0/2 (1)	16/39 (6) 41%
***Skeletal***							
Tall stature^b^	-	+	8/19	11/15	N.A.	2/2 (1)	22/38 (7) 58%
*Pectus* deformities	-	+	7/16 (3)	9/15	2/6	1/3	20/42 (3) 47.6%
Arachnodactyly	-	+	8/13 (6)	8/15	2/6	0/2 (1)	19/38 (7) 50%
Joint hypermobility	+ (BS 9/9)	+ (BS 6/9)	10/15 (4)	7/15 (BS > 5/9)	4/6	2/3	23/41 (4) 56%
Chronic Pain	+	+	N.A.	N.A.	N.A.	N.A.	2/2 (43)
Scoliosis	+	+	4/15 (4)	5/15	1/6	3/3	15/41 (4) 36.5%
Pes planus	-	-	11/15 (4)	8/15	1/6	2/2 (1)	22/40 (5) 55%
Club feet	-	-	0/15 (4)	5/15	1/6	0/2 (1)	6/40 (5) 15%
High arched palate	+	+	9/15 (4)	10/15	2/6	1/2 (1)	24/40 (5) 60%
***Cutaneous***							
Hyperelastic skin	+	+	0/13 (6)	N.A.	N.A.	N.A.	2/15 (30) 13%
Defective scaring	-	+	N.A.	2/15	1/6	0/2 (1)	4/25 (20) 16%
Easy bruising	+	+	N.A.	5/11 (4)	N.A.	0/2 (1)	7/15 (30) 46.6%
Striae distensae	+	-	8/15 (4)	3/15	1/6	0/2 (1)	13/40 (5) 32.5%
Hernia	+	-	6/17 (2)	10/15	N.A.	2/2 (1)	19/36 (9) 52.7%
***Other***							
Dural ectasia	+	N.A.	3/5 (14)	1/6 (9)	N.A.	0/2 (1)	5/14 (31) 35.7%

^a^Feature present/number of evaluated patients; in parenthesis number of patients with no data available; ^b^(>95th centile); ^c^at aortic root level, ectasia and/or aneurysm of ascending aorta, aortic arch and pulmonary artery were not ascertained; ^d^it is unclear whether these 5 patients were investigated. BS: Beighton score; N.A. not analyzed; MV: mitral valve; TAA: thoracic aortic aneurysm; MPA: main pulmonary artery aneurysm.

The incidence of arterial tortuosity is not well documented, given the limited number of patient analyzed. In particular, Boileau et al. found tortuosity in 3 out of 5 of the cases analyzed (14 patients were not investigated), and Linsday et al. in 1 out of 4 of the analyzed cases (11 patients not investigated) [16],[17]. Renard et al. identified tortuosity of the vertebral arteries in the patient with recurrent strokes, but it is unclear if the remaining patients were studied [18]. Leutermann et al. reported arterial tortuosity in both analyzed family members with the proband showing profound cerebral and cervical arterial tortuosity [19].

In our 57-year old proband and in her 34-year-old daughter aortic dissection or cardiovascular surgery did not occur and aortic aneurysms were not detected so far. Although, we are conscious that the cardiovascular phenotype of her daughter is incomplete, since no MRA was performed, echocardiography excluded at least aortic root involvement, the most common site of aneurysm in LDS4 [16]–[19]. Furthermore, in the proband only a brain, thoracic and abdominal MRA showed tortuosity and ectasia of the carotid, vertebral, and cerebral arteries and tortuosity of two segmental pulmonary arteries. Thus, the vascular features of our family strengthen the hypothesis of a milder vascular involvement in LDS4 compared to the other LDS types. Since a late-onset aneurysmatic vasculopathy remains possible, a periodical follow-up with vascular imaging was scheduled for the proband, and MRA study was highly recommended for her daughter.

Concerning cardiovascular surgery, Boileau et al. described 4 patients in the sixth decade, between them 3 were admitted for thoracic aortic dissection at 47 years, for TAA at 59 years and for MVP at 43 years (valve prosthesis) [17]. In Linsday et al., 2 patients were 50- and 61-year-old, and the first underwent VSARR [16]. Finally, Renard et al. described the oldest patient (69 years) who experienced a type A aortic dissection surgically treated at 60 years [18]. Three patients in sixth/seventh decade are also reported in this article, one of them had mitral valve prosthesis at 56 years for MVP [18]. Therefore, considering the age of our proband and the mean number of cardiovascular surgeries or major cardiovascular events in the patients over or in their sixth decade reported in literature, the vascular involvement in LDS4 can be even milder than described until now.

Since the tortuosity of the cerebral and neck arteries disclosed in our proband is a predisposing condition for aneurysm formation, we suggest that in LDS4 patients the vascular screening should not be restricted only to the thoracic arteries. An accurate evaluation of the brain and neck arteries, also if they might be involved only in a small percentage of patient, should be important in order to prevent severe cerebrovascular events.

Our proband and her 34-year-old daughter showed MVP, though with a low hemodynamic impairment, in agreement with the hypothesis of Renard et al. that pointed out a high rate of mitral valve disease, suggesting that this might be a signature feature of the disorder [18]. On the contrary, none of the family members reported by Leutermann et al. showed mitral valve impairment, suggesting that this hypothesis should be confirmed in larger series [19].

Concerning the musculoskeletal involvement (Table 2), Boileau et al. underlined a high rate of MFS signs, such as *pes planus* (73% of cases, 11/15), high arched palate (60% of the cases, 9/15), arachnodactyly (62% of cases, 8/13), and *pectus* deformities (44% cases, 7/16) [17]. Linsday et al., and Renard et al. confirmed the high prevalence of these signs: *pes planus* rate was 53% (8/15) and 17% (1/6) respectively; high arched palate rate was 67% (10/15) and 33% (2/6); arachnodactyly rate was 53% (8/15) and 33% (2/6). Finally, *pectus* deformities rate was 60% (9/15) in Lindsay et al., and 33% (2/6) in Renard et al. [16],[18]. The musculoskeletal signs observed in the family of Leutermann et al. were scoliosis (3/3), *pes planus* (2/2), high arched palate (1/2) and joint hypermobility (2/3) [19]. Joint hypermobility was also present in 67% (10/15) of the cases in Boileau et al., in 47% (7/15) of the cases in Linsday et al., and in 50% (3/6) of cases in Renard et al. [16]–[18]. Skeletal signs of MFS were present in all members of our family. A *marphanoid* habitus with tall stature for the familial target, *pectus excavatum*, high arched palate, and arachnodactyly were recognizable in the 34-year-old daughter of the proband. In the 39-year-old daughter tall stature for the familial target, *pes planus* and scoliosis treated with orthopedic corset were referred. The proband only showed scoliosis and high arched palate. Both patients of our family had a positive Beighton score. Articular pain associated with joint hypermobility was not reported in literature so far in patients with LDS4, whereas both our patients referred chronic generalized articular pain. In fact, the proposita and her daughter came to our attention not for cardiovascular complains, but mainly for joint hypermobility complications: chronic articular pain since young age in the proposita, and recurrent dislocations, either in the proposita and in her daughter.

In our study, the neurological involvement, i.e., dural ectasia, was monitored only in the proposita. While the significance of dural ectasia is well recognized in MFS [24],[25], its prevalence in LDS is less well documented [25],[26]. Kono et al. showed that the occurrence of dural ectasia in LDS1 and 2 was significantly higher than in controls with a frequency that varied from 40 to 70% [27]. The authors suggested that dural ectasia has the potential to become a diagnostic criterion for LDS. In support of this hypothesis, a recent comprehensive study demonstrated that dural ectasia occurs in LDS with a similar frequency and severity as in MFS [28]. Concerning LDS4 patients, Boileau et al. found dural ectasia in 3 out of 5 patients, Linsday et al. in 1 out of 10, whereas Leutermann et al., did not find this sign, and Renard et al. did not investigate it [16]–[19]. Given the fact that the incidence data of dural ectasia are limited to a small number of LDS4 patients (5/14, with 31 patients not analyzed), its presence should be studied in larger series of patients.

## Conclusions

In conclusion, we suggest that LDS4 should be considered in patients of all ages with variable but mostly mild LDS-like phenotypes and/or with sparse signs of MFS and related connective tissue disorders with negative *TGFBR1/2* and *SMAD3* molecular tests, even though TAA and major cardiovascular events are absent, as it was for our proband. Molecular diagnosis allows the early identification of patients and relatives at risk of major cardiovascular complications. Further studies are needed to better delineate the clinical phenotype of LDS4 and search for new therapeutic options.

### Consent

Written informed consent was obtained from patients for publication of this article and accompanying images. A copy of the written consent is available for review by the Editor-in-Chief of this journal.

## Abbreviations

CTD: Connective tissue disorders

ECG: Electrocardiogram

EF: Ejection fraction

LDS: Loeys-Dietz syndrome

MFS: Marfan syndrome

MRA: Magnetic resonance angiography

MRI: Magnetic resonance imaging

MVP: Mitral valve prolapse

NMD: Nonsense-mediated RNA decay

NSAIDs: Nonsteroidal anti-inflammatory drugs

PTC: Premature termination codon of translation

RT-PCR: Reverse–transcriptase polymerase chain reaction

SNP: Single nucleotide polymorphism

TAAD: Thoracic aortic aneurysm and dissection

VSARR: Valve sparing aortic root replacement

## Competing interests

There are no competing interests.

## Authors’ contributions

MR, NC, SQ performed the molecular analyses. CD, MV, PCP, and MC made the clinical evaluation of the patients and performed the genetic counseling and follow-up. RM performed the MRA study. MR, NC and CD researched the literature, reviewed and prepared the manuscript. MR, NC and MC edited and coordinated the manuscript. All of the authors discussed, read, and approved the manuscript.
